# The prevalence of eating behaviors among Canadian youth using cross-sectional school-based surveys

**DOI:** 10.1186/1471-2458-14-323

**Published:** 2014-04-07

**Authors:** Heather G Lillico, David Hammond, Steve Manske, Donna Murnaghan

**Affiliations:** 1 School of Public Health & Health Systems, University of Waterloo, 200 University Avenue West, Waterloo, Ontario N2L 3G1, Canada; 2 Propel Centre for Population Health Impact, University of Waterloo, 200 University Ave West, Waterloo, Canada; 3 School of Nursing, University of Prince Edward Island, 550 University Ave, Charlottetown, Canada

**Keywords:** Obesity, Adolescent, Eating habits

## Abstract

**Background:**

Obesity is a growing public health concern in Canada. Excess weight is particularly a concern among youth given that obesity in youth predicts obesity in adulthood. Eating behaviors, both inside and outside the home have been associated with increased risk of obesity; however, there is little data among Canadian youth to monitor trends.

**Methods:**

The School Health Action, Planning and Evaluation Surveys (SHAPES) were administered in schools. Our study examined 20, 923 students (grades 5-12) from four regions in Canada. The regions were Hamilton and Thunder Bay (both in Ontario), the Province of Prince Edward Island, and the Province of Quebec.

**Results:**

Consuming breakfast daily was reported by 70% of grade 5-8 students, and 51% of grade 9-12’s. Among students in grade 9-12, 52% reported eating with family members daily, compared with 68% in grade 5-8. Just over half of students in grade 5-8, and 70% in grade 9-12 reported eating at a fast-food place once a week or more. Among grade 5-8 students 68% reported eating in front of the television at least once per week, compared to 76% in grade 9-12. Obese students were more likely to watch TV while eating, and less likely to eat with a family member and eat breakfast.

**Conclusions:**

The findings suggest that only a modest proportion of youth report dietary patterns that have previously been associated with healthy eating and reduced risk of obesity. Later adolescence may be a critical time for intervention in health-related behaviors.

## Background

The prevalence of overweight and obese youth is a growing public health concern in Canada. According to the Canadian Health Measures Survey 2009-2011, approximately 20% of youth aged 12-17 are overweight, and 10% are obese [1]. Excess weight is particularly a concern among children and youth as obesity during this period is associated with obesity during adulthood [2]. Excess weight is also a risk factor for cardiovascular disease, type 2 diabetes, high blood pressure, osteoarthritis, gallbladder disease, and certain cancers [3]. There is evidence to suggest that certain eating patterns affect diet quality, energy intake and/or weight.

### Skipping breakfast

Excess energy intake is a primary risk factor for obesity. Certain patterns of eating, such as skipping breakfast, have been associated with energy intake and increased weight gain [4-7]. Breakfast skipping is thought to affect weight status by increasing energy intake; not eating in the morning may lead to increased feelings of hunger and consequently overeating later in the day [6]. In addition, those who skip breakfast are more likely to consume snacks that are higher in fat, and obtain more calories from fat in their daily diet than those who eat breakfast [7]. One factor that has been shown to increase the likelihood of adolescents consuming breakfast is having a parent present in the morning [8].

### Eating meals with family members

Eating meals with family members has also been associated with healthier diet. A systematic review in 2008 reported that 25-57% of adolescents ate meals with their family five or more times per week, and that the number of family meals decreases with age [9]. A five-year longitudinal study of middle-school and high-school students found that eating family meals was associated with improved dietary intake, and better psychological well-being; however, no consistent associations were observed with obesity [10]. The literature is inconsistent as to the effect of family meals on weight status. In a longitudinal Canadian study, family meals were associated with a lower BMI in female adolescents, but not males [11]. In another study an inverse association was found between eating meals together and overweight status cross-sectionally; however, after three years of follow-up there was no longitudinal association between the likelihood of becoming overweight and consuming meals with family members [12]. In another study, family dinners were associated with an increase in servings of fruits or vegetables, but having the television on during these family meals was associated with a decrease in those foods, thereby negating some of the benefits of eating together [13].

### Fast-food consumption

Fast-food consumption is a prevalent eating behavior that affects weight status. Estimates from a national survey conducted in 2004 indicate that one third of Canadian teenagers aged 14-18, and one quarter of all Canadians consumed food at a fast-food outlet in the last 24 hours [14]. While not all food consumed outside of the home is of poor nutritional quality, restaurants generally offer foods that have larger portions, and are higher in calories and fat [15]. Fast-food consumption is associated with increased energy intake, and increased calorie consumption from fat [16-18]. Increased consumption of sugar sweetened beverages and fried potatoes is also associated with eating fast-food [17,19]. The literature suggests that the odds of becoming overweight or obese increase with greater fast-food consumption [20]. Few Canadian studies address fast-food consumption among youth. One cross-sectional study found that obese individuals consumed more fast-food than non-obese children and adolescents, and that percent body fat was positively correlated with consuming food outside of the home [21]. Obese children and adolescents also consumed more grain products, and sugar sweetened drinks, than non-obese individuals. Expanding on that, another study found that grades 6-8 students in Ontario, Canada consumed higher amounts of energy, meats and alternatives and “other” foods (according to Canada’s Food Guide) when they ate lunch at restaurants/fast-food outlets compared to eating at home/school [22]. It is also important to note that increased fast-food consumption is associated with decreased fruit and vegetable consumption [16,17,23].

### Eating in front of the television

Eating in front of the television has also been associated with diet quality and weight status. Diet quality is lower among children who eat in front of the TV more often; they eat less fruits and vegetables, have higher energy intakes from fat and sugar, and consume more soft-drinks [24-27]. In addition, individuals consume more calories and larger portions while eating in front of the TV [28]. Not surprisingly, watching television while eating is positively associated with BMI [29]. Liang and colleagues found that watching TV by itself, and eating while watching television were both independently associated with being overweight [24].

Given the increasing prevalence of obesity, there is a need to monitor changes in eating patterns among youth. Specifically, it is important to examine behaviors that have been shown to affect diet quality and weight status to better understand their relationship with obesity.

To date, however, there is very little evidence among Canadian youth with which to track changes over time. The specific research objectives of the current paper are to: 1) examine the frequency of the four eating behaviors: eating breakfast, eating with an adult family member, eating at a fast-food place or restaurant, and eating in front of the television, and 2) explore the extent to which these eating behaviors differ across socio-demographic groups and geographic regions.

## Methods

### Participants

Students in grade 5-12 who completed the School Health Action, Planning and Evaluation System (SHAPES) were included in the current study [30]. SHAPES surveys were conducted in four regions in Canada: 1) Hamilton, Ontario, 2) Thunder Bay, Ontario, 3) the Province of Prince Edward Island (PEI), and 4) the Province of Quebec. Respondents for whom age, sex or grade was missing or inconsistent were excluded from the analysis (n = 295). The Hamilton data set included grades 9-12; questionnaires were administered in 8 schools and data were collected from roughly 5,000 students. Thunder Bay data included students in grades 9-12 and were collected in 9 schools with approximately 3,000 students. The PEI data set included grades 5-12, with over 6,000 students and was collected in 54 schools, and the Quebec data set included grades 5 & 6, and secondary I-V, and was collected in 140 schools from approximately 9,000 students. The Healthy Eating Module (which contained the outcomes of interest) was not administered to all students, so 7722 participants were excluded from current data analyses for this reason.

### Instruments

Data from the SHAPES were used for this study. Topics include tobacco use, physical activity, healthy eating and mental fitness [31]. Tailored results from the in-class, self-administered, machine readable questionnaire are reported back to schools after data collection [32].

Demographic variables included age (in years), grade (5-12) and sex. Self-reported height (in centimetres) and weight (in kilograms) were collected and used to calculate body mass index (BMI). The World Health Organization 2007 BMI cut-off values were used to categorize individuals into underweight, healthy weight, overweight and obese [33]. Height and weight were not asked for grades 5-8 in PEI, and grades 5-6 in Quebec and so BMI could not be computed for this sub-set of respondents.

The Healthy Eating Module asked respondents about various eating-related behaviors, including: “In a usual school week (Monday to Friday), how many times do you do the following…?” “Eat breakfast”; “Eat foods purchased at a fast-food place or restaurant”; “Eat meals while watching television”; and “Eat meals with at least one adult family member.” The response options for Hamilton, Thunder Bay and PEI data collection were: none, less than once a week, 1 time, 2 times, 3 times, 4 times, 5 times, and 6+ times per week. Quebec survey responses were: none, 1 time per week, 2 times, 3 times, 4 times, and 5+ times per week. Responses were re-categorized into none, once per week or less than once per week, 2 times, 3 times, 4 times, and 5+ times per week to standardize across surveys.

### Procedure

Data for Hamilton were collected between December 2009 and April 2010, and Thunder Bay data were collected in December 2009. Data from PEI were collected November 2010 until April 2011 and Quebec data were collected between January 2011 and May 2011. Ethics approval was provided by Research Ethics Boards at the University of Waterloo and the University of Prince Edward Island, and from appropriate School Board Ethics committees. The process for consent differed depending on participant age. For students aged 14 and older, active information/passive consent was used. School personnel mailed information letters to parents. The information letter explained the nature and rationale of the survey and provided clear opportunities to decline their child’s participation. Parents could call a toll-free number to ask questions about the survey, or decline participation. Permission to participate was granted if parents did not contact project/school staff. Passive consent was used to decrease burden on the schools, and the level of risk involved was deemed minimal. If parents declined participation on their child’s behalf, then they could not participate. For students under the age of 14, active consent was used. Students brought a letter home which provided the nature and rationale of the survey and clearly stated that participation is voluntary. Parents were asked to indicate if they provided permission for their child to participate. Only students with returned, signed permission forms were able to participate. On the day of survey administration teachers read a standardized script that provided information about the surveys and confidentiality procedures. Students were also told that their participation was voluntary and they could refuse to participate at any time without penalty. Students who did not wish to participate were given an alternate activity. No compensation was provided for participation.

### Data analysis

Descriptive statistics are reported for each of the four eating behaviors and the association between the four outcomes was examined using Pearson correlations. Separate logistic regression models were fitted to examine predictors of the four eating behaviors. Logistic regression models were used because the data was not normally distributed and did not meet assumptions for ordinal regression. Each of the outcome behaviors were dichotomized. The outcomes of eating breakfast and eating with an adult family member were both recategorized into five or more times per week and all other frequencies per week. The outcomes of eating at a fast-food place or restaurant, and eating in front of the television were recategorized into zero times per week and all other frequencies per week. Regression models were stratified by grade because BMI was not uniformly available for grades 5-8. Additionally, models were stratified by grade given the differenes between between primary and secondary school settings, including with respect to food environments within schools. The models run with younger students (grades 5-8) included three covariates: age, gender and survey location. The models run with older students (grades 9-12) included four covariates: age, gender, BMI level and survey location. Age and grade were not entered into the same model because they were highly correlated. Odds ratios are reported in all cases. Analyses were conducted using SPSS (version 20, 2011, IBM Corp., Armonk, NY).

## Results

### Sample characteristics

Sample characteristics are shown in Table 1. As noted above, Thunder Bay and Hamilton samples did not include grade 5-8 students. In Quebec, secondary schools end at Secondaire V, equivalent to Grade 11.

**Table 1:** Sample characteristics N = 20,923

	**PEI n = 1 417**	**Quebec n = 12 246**	**Hamilton n = 2 541**	**Thunder bay n = 1 499**	**PEI n = 1 812**	**Quebec n = 1 048**
	**Grade 5-8**		**Grade 9-12**			
**Age**						
11 or under	46.5%	67.9%	-	-	-	-
12	25.9%	23.3%	-	-	-	0.1%
13	23.7%	5.8%	0.3%	1.9%	0.9%	-
14	3.8%	2.5%	23.3%	32.4%	20.7%	16.5%
15	0.1%	0.4%	25.1%	32.5%	26.8%	32.5%
16	-	-	24.7%	24.1%	22.8%	32.2%
17 or older	-	-	26.5%	9.1%	28.8%	18.7%
**Mean**	11.9	11.4	15.5	15.1	15.6	15.5
**(SD)**	(0.92)	(0.75)	(1.12)	(1.00)	(1.14)	(0.98)
**Grade**						
5	29.6%	50.5%	-	-	-	-
6	19.8%	39.7%	-	-	-	-
7	24.0%	4.9%	-	-	-	-
8	26.6%	4.9%	-	-	-	-
9	-	-	28.9%	35.4%	24.0%	36.3%
10	-	-	24.3%	33.0%	27.3%	30.7%
11	-	-	24.7%	23.8%	24.6%	33.0%
12	-	-	22.0%	7.7%	24.2%	-
**Gender**						
Female	49.0%	49.7%	50.1%	49.5%	47.5%	50.7%
Male	51.0%	50.3%	49.9%	50.5%	52.5%	49.3%
**BMI**^**a**^						
Underweight	-	-	1.6%	1.3%	1.0%	1.3%
Healthy weight	-	-	56.6%	53.4%	56.1%	62.5%
Overweight	-	-	11.0%	13.7%	15.3%	11.6%
Obese	-	-	4.8%	5.3%	7.4%	4.5%
Not Stated	-	-	26.1%	26.3%	20.1%	20.1%

^a^BMI was not asked of grade 5-8 students.

### Prevalence of eating behaviors

Tables 2 and 3 show the prevalence of four eating behaviors among students in grade 5-8, and those in grade 9-12 for each of the four regions.

**Table 2:** Prevalence of four eating behaviors in various geographic regions (Grade 5-8)

**In a usual week (Monday to Friday), how often do you:**	**PEI n = 1 417**	**Quebec n = 12 246**	**Total n = 13 663**
**Eat breakfast**			
None	3.9%	3.4%	3.5%
Once/week or less	9.2%	9.5%	9.5%
2 times/week	5.7%	4.1%	4.2%
3 times/week	7.2%	5.0%	5.3%
4 times/week	6.5%	7.3%	7.2%
5+ times/week	67.4%	70.7%	70.4%
**Eat meals with at least one adult family member**			
None	8.0%	4.5%	4.9%
Once/week or less	8.0%	5.3%	5.6%
2 times/week	5.0%	5.0%	5.0%
3 times/week	6.8%	7.2%	7.2%
4 times/week	9.1%	9.2%	9.2%
5+ times/week	63.1%	68.8%	68.2%
**Eat foods purchased at a fast-food place/restaurant**			
None	43.7%	44.8%	44.7%
Once/week or less	42.0%	37.3%	37.8%
2 times/week	8.8%	10.0%	9.9%
3 times/week	2.8%	3.3%	3.3%
4 times/week	0.6%	1.4%	1.3%
5+ times/week	2.2%	3.1%	3.0%
**Eat meals while watching television**			
None	30.3%	32.2%	32.0%
Once/week or less	27.9%	21.6%	22.3%
2 times/week	11.6%	13.3%	13.1%
3 times/week	9.7%	10.3%	10.2%
4 times/week	5.9%	6.1%	6.0%
5+ times/week	14.5%	16.5%	16.3%

**Table 3:** Prevalence of four eating behaviors in various geographic regions (Grade 9-12)

**In a usual week (Monday to Friday), how often do you:**	**Hamilton n = 2 541**	**Thunder bay n = 1 499**	**PEI n = 1 812**	**Quebec n = 1 408**	**Total n = 7 260**
**Eat breakfast**					
None	12.2%	11.7%	9.5%	7.4%	10.5%
Once/week or less	15.8%	14.4%	12.1%	3.6%	12.2%
2 times/week	9.5%	7.6%	9.1%	5.9%	8.3%
3 times/week	10.1%	9.6%	9.9%	7.4%	9.4%
4 times/week	8.8%	8.4%	6.7%	9.2%	8.3%
5+ times/week	43.7%	48.3%	52.7%	66.5%	51.3%
**Eat meals with at least one adult family member**					
None	8.7%	7.5%	8.1%	4.8%	7.5%
Once/week or less	11.4%	12.3%	10.0%	4.0%	9.8%
2 times/week	9.2%	8.4%	7.1%	4.4%	7.6%
3 times/week	11.7%	11.1%	10.5%	8.1%	10.6%
4 times/week	12.8%	12.5%	11.4%	13.3%	12.5%
5+ times/week	46.2%	48.2%	52.9%	65.3%	52.0%
**Eat foods purchased at a fast-food place/restaurant**					
None	24.9%	32.8%	28.9%	38.9%	30.2%
Once/week or less	45.0%	45.8%	40.7%	39.2%	43.0%
2 times/week	13.9%	11.3%	13.3%	13.1%	13.0%
3 times/week	6.9%	4.4%	7.3%	4.2%	5.9%
4 times/week	3.4%	1.8%	3.1%	1.6%	2.7%
5+ times/week	6.0%	4.0%	6.8%	3.0%	5.2%
**Eat meals while watching television**					
None	20.9%	22.5%	24.0%	33.1%	24.4%
Once/week or less	27.8%	25.9%	23.2%	20.1%	24.8%
2 times/week	16.3%	15.5%	13.6%	12.5%	14.7%
3 times/week	12.7%	14.5%	13.8%	10.3%	12.8%
4 times/week	6.3%	6.3%	8.5%	6.5%	6.9%
5+ times/week	16.0%	15.2%	16.9%	17.6%	16.4%

#### Eating breakfast

A total of 70% of students in grade 5-8 reported eating breakfast 5 times per week or more, and fewer than 5% of students reported not eating breakfast at all during the week. Among grade 9-12 students 50% of students reported eating breakfast 5 times per week or more, and roughly 10% reported not eating breakfast at all in a typical week.

#### Eating with an adult family member

Just over two thirds of students in grade 5-8 reported eating meals with at least one adult family member 5 or more times per week, compared with roughly 5% who reported not eating with an adult at all during the week. Among students in grade 9-12, just over half reported family meals 5 or more times during the week, and approximately 8% reported not eating any meals with an adult during the week.

#### Eating at a fast-food place or restaurant

Just over half (55%) of students in grade 5-8 reported consuming food at a fast-food place or restaurant once a week or more, and less than half (45%) reported not eating at a fast-food place or restaurant during the week. Among grade 9-12 students 70% reported eating fast-food at least once a week, and 30% said they consumed no fast-food during the week.

#### Eating in front of the television

Almost 70% of students in grade 5-8 reported eating in front of the television at least once per week, while about one third reported no meals in front of the TV per week. Students in grade 9-12 reported eating in front of the television in about 75% of cases, and 25% reported not eating in front of the television during the week.

#### Correlations between eating behaviors

Correlations were examined between the four eating behaviors. Eating breakfast was negatively associated with eating at a fast-food place or restaurant (r = -0.066, p < 0.001), and eating while watching television (r = -0.038, p < 0.001). Eating breakfast was positively associated with eating with at least one adult family member (r = 0.326, p < 0.001). Eating at a fast-food place or restaurant was positively associated with eating while watching television (r = 0.221, p < 0.001), but negatively associated with eating with an adult family member (r = -0.070, p < 0.001). Eating while watching television was negatively associated with eating with an adult family member (r = -0.081, p < 0.001). When the analysis was stratified by grade level (grade 5-8 vs. grade 9-12), the pattern of results were the same.

### Correlates of eating behaviors

Tables 4 and 5 show the results of the logistic regression models used to examine correlates of eating behaviors, while adjusting for other factors.

**Table 4:** Correlates of eating behaviors (breakfast & family meals)

	**Eating breakfast**		**Eating with an adult Family member**	
	**Grade 5-8**	**Grade 9-12**^**a**^	**Grade 5-8**^**a**^	**Grade 9-12**^**a**^
**Adjusted R**^**2b**^	.013	0.062	0.003	0.038
**Sex**				
Female (ref) vs. Male	1.37^***^	1.60^***^	0.96	1.00
**Age**	0.84^***^	0.90^***^	0.95^*^	0.86^***^
**Site**				
PEI (ref) vs. Quebec^c^	1.089	1.80^***^	1.26^***^	1.66^***^
PEI vs. Hamilton	-	0.70^***^	-	0.76^***^
PEI vs. TBay	-	0.81^**^	-	0.77^***^
Quebec (ref) vs. Hamilton	-	0.39^***^	-	0.46^***^
Quebec vs. TBay	-	0.45^***^	-	0.47^***^
Hamilton (ref) vs. TBay	-	1.16^**^	-	1.02
**BMI**^**d**^				
Healthy Weight (ref) vs. Underweight	-	1.20	-	1.97^**^
Healthy Weight vs. Overweight	-	0.95	-	0.97
Healthy Weight vs. Obese	-	0.81^*^	-	0.83
Healthy Weight vs. Not Stated	-	0.73^***^	-	0.84^**^
Not Stated (ref) vs. Underweight	-	1.65^*^	-	2.35^***^
Not Stated vs. Overweight	-	1.30^**^	-	1.16
Not Stated vs. Obese	-	1.11	-	0.99
Underweight (ref) vs. Overweight	-	0.79	-	0.49^**^
Underweight vs. Obese	-	0.67	-	0.42^***^
Overweight (ref) vs. Obese	-	0.85	-	0.85

^*^P < 0.05 ^**^P < 0.01 ^***^P < 0.001. Bolded type denotes significance of at least <0.05.

^a^Odds ratios are shown for binary logistic regressions adjusting for sex, age, site and BMI (except for grade 5-8).

^b^The R^2^ value presented is a Nagelkerke R^2^.

^c^Grades 5-8 only have data available from PEI and Quebec site locations.

^d^BMI was unavailable in grade 5-8 students.

**Table 5:** Correlates of eating behaviors (fast-food & television)

	**Fast-food place or restaurant**		**Eating in front of the TV**	
	**Grade 5-8**^**a**^	**Grade 9-12**^**a**^	**Grade 5-8**^**a**^	**Grade 9-12**^**a**^
**Adjusted R**^**2b**^	0.005	0.031	0.001	0.020
**Sex**				
Female (ref) vs. Male	1.27^***^	1.42^***^	1.07	1.16^**^
**Age**	1.05^*^	1.11^***^	1.00	1.00
**Site**				
PEI (ref) vs. Quebec^c^	0.98	0.65^***^	0.92	0.65^***^
PEI vs. Hamilton	-	1.25^**^	-	1.23^**^
PEI vs. TBay	-	0.89	-	1.11
Quebec (ref) vs. Hamilton	-	1.94^***^	-	1.89^***^
Quebec vs. TBay	-	1.37^***^	-	1.71^***^
Hamilton (ref) vs. TBay	-	0.71	-	0.90
**BMI**^**d**^				
Healthy Weight (ref) vs. Underweight	-	0.77	-	0.71
Healthy Weight vs. Overweight	-	0.95	-	1.04
Healthy Weight vs. Obese	-	1.14	-	1.35^*^
Healthy Weight vs. Not Stated	-	0.97	-	0.90
Not Stated (ref) vs. Underweight	-	0.80	-	0.79
Not Stated vs. Overweight	-	0.98	-	1.15
Not Stated vs. Obese	-	1.17	-	1.50^**^
Underweight (ref) vs. Overweight	-	1.23	-	1.45
Underweight vs. Obese	-	1.47	-	1.89^*^
Overweight (ref) vs. Obese	-	1.20	-	1.31

^*^P < 0.05 ^**^P < 0.01 ^***^P < 0.001. Bolded type denotes significance of at least <0.05.

^a^Odds ratios are shown for binary logistic regressions adjusting for sex, age, site and BMI (except for grade 5-8).

^b^The R^2^ value presented is a Nagelkerke R^2^.

^c^Grades 5-8 only have data available from PEI and Quebec site locations.

^d^BMI was unavailable in grade 5-8 students.

#### Eating breakfast

Among grade 5-8 students, males (p < 0.001), and younger students (p < 0.001) were significantly more likely to eat breakfast. The model with students in grade 9-12 revealed the same pattern for age (p < 0.001) and sex (p < 0.001). The associations for site showed that those in Quebec were more likely to eat breakfast than those in Hamilton (p < 0.001), PEI (p < 0.001) and Thunder Bay (p < 0.001). Those in PEI were more likely than those in Thunder Bay to eat breakfast (p = 0.004). Students in Thunder Bay were more likely than those in Hamilton to eat breakfast (p = 0.03). Students who were obese were less likely to eat breakfast compared to healthy weight individuals (p = 0.046). Those who were underweight (p = 0.020), healthy weight (p < 0.001), or overweight (p < 0.001) were more likely to eat breakfast than those who did not state their height and weight.

#### Eating with an adult family member

Among students in grade 5-8, older students (p = 0.017), and those living in PEI (p < 0.001) were less likely to eat with an adult family member. Students in grade 9-12 were less likely to eat with an adult family member as they got older (p < 0.001). Students living in Quebec were more likely to eat with an adult family member compared to those in Hamilton (p < 0.001), Thunder Bay (p < 0.001), and PEI (p < 0.001). Additionally, those in PEI were more likely than those in Thunder Bay (p < 0.001) and Hamilton (p < 0.001) to eat with a family member. Students who were underweight were more likely to eat with an adult compared to healthy weight (p = 0.003), overweight (p = 0.002), and obese (p < 0.001) students. Additionally, underweight (p < 0.001), and healthy weight students (p = 0.002) were more likely to eat with an adult than those who did not state their height and weight.

#### Eating at a fast-food place or restaurant

Among grade 5-8 students, males (p < 0.001), and older students (p = 0.023) were more likely to consume food at a fast-food place or restaurant. The model run with students in grades 9-12 revealed the same pattern for sex (p < 0.001) and age (p < 0.001). With regards to site, students in PEI (p < 0.001), Hamilton (p < 0.001), and Thunder Bay (p < 0.001) were more likely to consume fast-food than those in Quebec. There were no significant correlations with respect to BMI.

#### Eating in front of the television

In the model with students in grades 5-8 no values were significant. In the model with students in grades 9-12, males were more likely than females to eat in front of the television (p = 0.009). Additionally, those in those in PEI (p < 0.001), Hamilton (p < 0.001), and Thunder Bay (p < 0.001) were more likely than students in Quebec to eat in front of the television. Those in Hamilton were also more likely than those in PEI to eat in front of the television (p = 0.006). Obese students were also more likely than underweight (p = 0.014), healthy weight (p = 0.026), and those who did not state their height and weight (p = 0.004) to eat in front of the television.

Models were run for each of the four dichotomized eating behavior outcomes to compare students in grade 5-8 with those in grade 9-12. Students in grade 9-12 were less likely to eat breakfast (OR = 0.61, p < 0.001), and eat with an adult family member (OR = 0.69, p < 0.001) than those in grade 5-8. Those in grade 9-12 were more likely to eat at a fast-food place or restaurant (OR = 1.58, p < 0.001), and eat in front of the television (OR = 1.18, p < 0.001) than those in grade 5-8.

## Discussion

The current study examined patterns of eating behaviors among a large sample of Canadian youth. The findings indicate that almost one third of students in grades 5 to 8 and one half of grades 9-12 students skipped breakfast at least once per week. These estimates are higher than US data collected between 1999 and 2006 from the National Health and Nutrition Examination Survey, in which 20% of 9-13 year olds and 32% of 14-18 year olds reported skipping breakfast [34]. Additional research is required to examine whether the higher prevalence of breakfast skipping in the current study reflects differences between geographic regions or a change in trends over time. Present day breakfast skipping may indicate increased busyness in the morning, or a conscious effort to avoid eating breakfast.

Slightly more than half of students in grade 9-12 from the current study reported eating with family members 5 or more times per week, while a review from 2008 indicated that between 25%-57% of adolescents eat meals with family members this often [9]. One Canadian study found the prevalence of engaging in family meals every night to be 62%, but this data was from a single city, whereas the current study used a larger sample across multiple regions, which may offer greater representativeness [11]. Another Canadian study in 2005 using students from Nova Scotia found the prevalence of eating family meals 5 or more times per week to be 59% among grade 5 students, compared to 68% in the current study for grade 5-8 students [35].

Results from the current study revealed that 18% of students in grade 5-8, and 27% of those in grade 9-12 ate fast-food twice per week or more. Canadian data from 2009 estimated that 38% of youth aged 13-23 consume fast-food twice a week or more [36]. The current study’s results are slightly lower than expected. American data suggests that 75% of teenagers consume fast-food at least once a week, compared to estimates in the current study of 55% of students in grade 5-8, and 70% in grade 9-12 eating at a fast-food place or restaurant once a week or more [37]. These data indicate a lower consumption of fast-food in Canada versus America among the youth population, and a lower prevalence than previously thought among Canadian data.

The findings indicate that 16% of students in grade 5-8 and 9-12 ate meals in front of the television 5 times per week or more. This estimate is similar to previous data collected in Canada, but significantly less than in the US, in which estiamtes have ranged from 30% to 64% of youth who report eating in front of the TV on a daily basis [24,27,38]. One possible reason for this may be a fundamental difference in the way Canadians and Americans view meal times. Canadians may place more importance on having conversations during meal times, and not bringing media to the dinner table.

To our knowledge, no other study has examined differences in these four eating behaviors across various geographic regions in Canada. Considering how distinct the survey locations were, the results across regions were strikingly similar, with a few notable exceptions. Students in Quebec were more likely than other regions to eat with an adult family member, and consume breakfast. One possible reason for increased breakfast consumption in certain regions may be due to the presence of breakfast programs. However, it is unclear based on the number of programs offered in each Province, and the population size, whether this is the case. Breakfast clubs of Canada has 297 programs in Quebec, including club des petits dejeuners du Quebec. PEI only has one breakfast program, and Ontario has 386, 51 of which are in the Hamilton region, and 31 are in the Northwest (which includes Thunder Bay) [39]. Other reasons for a higher prevalence of these behaviors in Quebec may be related to a culture that values family meal time.

The results from the current study indicate that youth who are obese are less likely to eat breakfast than students who have a healthy weight. Findings from past research indicates that skipping breakfast is associated with weight gain [4,5,40]. Diet quality was not examined in this study, so it is unclear whether this is the mechanism through which not eating breakfast affects weight status.

Previous research is inconsistent regarding the association between weight status and eating meals with family members. The current study found that students who were obese, overweight, or of a healthy weight were less likely to eat meals with family members than underweight individuals. Eating family meals has been shown to have a positive effect on diet quality, and may increase family connectedness, and health-promoting behaviors [41]. It is unclear from this study whether people who are obese choose to eat less with their family, or whether engaging in fewer family meals leads to obesity possibly through deterioration of diet quality and/or health-promoting behaviors.

The results from the current study are inconsistent with the literature on fast-food consumption among youth: students who were obese were not more likely to consume fast-food than non-obese individuals in the current study [20,21,23]. Previous literature has found an association between consumption of fast-food and obesity. Possible reasons for this discrepancy may be due to the nature of the questions asked in the current study. Participants were asked about the number of times they ate at a fast-food place or restaurant during the week, but it is unclear what type of food they were consuming and the nutritional value of this food. Additionally, the analysis in the current study examined patterns of weekday consumption (Monday to Friday); weekend consumption of fast-food may depict a different pattern.

With respect to watching TV while eating, the current study found that obese students were more likely to engage in this behavior compared to those who were underweight or of a healthy weight, which is consistent with the literature [24,29]. Possible reasons for the association between weight status and watching TV while eating include increased exposure to advertisements of unhealthy food which affects purchasing behavior, the presence of television preventing dialogue between families about healthy eating practices, and mindless eating in front of the television resulting in larger portions consumed [24,26].

Differences in the four eating behaviors emerged between age groups; older students (grade 9-12) were less likely to eat breakfast, and eat meals with at least one adult family member. Breakfast consumption may decrease with age for a variety of reasons such as lack of time, or to intentionally decrease caloric intake. Eating meals with family members may lessen as a student gets older due to lack of compatibility with adults’ schedules, or increased autonomy. Older students were also more likely to eat at a fast-food place or restaurant. Adolescence is a time for fostering independence which may be exerted by purchasing one’s own food, likely fast-food for its convenience and low price [42]. Older students were also more likely to eat meals in front of the television. Possible reasons for this include a lack of parental presence during meal-time or a greater interest in media-related activities than the younger age group.

Certain gender differences in the current study were inconsistent with past research. A study by Neumark-Sztainer and colleagues found that males were more likely to eat with family members than females [43]. Among all ages in the current study there were no differences between sexes with respect to eating with an adult family member.

The present study found that males were more likely to consume food at a fast-food place or restaurant, and eat while watching TV (only among grade 9-12 students), a finding that is consistent with past data [17,18,25]. The current study also found that females were more likely than males to skip breakfast among all age ranges, which is consistent with past Canadian, and International research [34,44]. Possible reasons include weight and/or body image concerns; girls are more likely to engage in dieting practices that involve calorie restriction and meal skipping [34,44]. Research suggests however that this is an ineffective dieting practice, as people who skip breakfast are more likely to overeat later in the day [6].

### Strengths and limitations

This study exhibits several strengths. It boasts a large sample size drawn from four distinct geographical areas. The large sample size of the current study provided considerable power to detect small statistical differences. For example, to detect a bivariate difference in an outcome such as “frequency of eating at a fast food place or restaurant”, the current study had sufficient power to detect a 2.4% difference for a 2-sided t-test, where α = .05 and Beta = .80.

In addition, the value of having common measures and indicators cannot be underestimated [45,46]. Recent efforts have helped promote more consistent reporting and measurement for youth health [47-50].

In addition to the strengths, the current study has limitations. The current study is cross-sectional in nature, therefore, no causal inferences should be made regarding associations between variables. All analyses are based on self-completed surveys and self-reported data. The limitations of self-reported height and weight have been identified in previous literature [51,52]. However, the measure of height and weight used by SHAPES has been validated and the project has been conducted in over 3000 schools with over 500,000 students since the year 2000. Additionally, with the measures used correlations between self-reported measures of height and weight and actual measures were significant [53]. Another limitation is that even though surveys were anonymous, certain sensitive questions may have been influenced by social desirability bias. For example, students may feel uncomfortable admitting that they consume fast-food every day of the week.

Finally, while most survey items in different regions were consistent, in a few instances, response categories needed to be collapsed to ensure comparability. For example, behaviors of once per week and less than once per week were combined, which decreased the specificity of responses.

## Conclusions

The current study adds to the evidence base on patterns of eating among Canadian youth. Students categorized as obese were significantly more likely to report watching TV while eating, and less like to report eating with an adult family member, and eating breakfast. Future research should focus on identifying trends over time and providing greater insight into the causal associations between eating behaviors and obesity. In addition, the current study found that older students were less likely to eat breakfast, and eat meals with a family member, and more likely to consume fast-food and eat in front of the television. Late adolescence seems to be a critical time for certain eating behaviors, and interventions aimed at promoting healthy behaviors during this time may be beneficial.

## Competing interests

The authors declare that they have no competing interests.

## Authors’ contributions

HGL and DH performed data analysis and drafted the manuscript. SM and DM contributed to manuscript preparation. All authors read and approved the final manuscript.

## Pre-publication history

The pre-publication history for this paper can be accessed here:

http://www.biomedcentral.com/1471-2458/14/323/prepub
